# 304. Global Incidence of Viral Lower Respiratory Tract Disease (LRTD) Episodes and Hospitalizations (2010–2021)

**DOI:** 10.1093/ofid/ofae631.094

**Published:** 2025-01-29

**Authors:** Ekaterina Maslova, Malin Fageras, Kate W Gillespie, Lisa White, Quinn Raferty, Andrei Oros, Pratik Sinha

**Affiliations:** AstraZeneca, London, England, United Kingdom; AstraZeneca, London, England, United Kingdom; Insitute for Health Metrics and Evaluation, Seattle, Washington; Model Health ltd, Oxford, England, United Kingdom; Insitute for Health Metrics and Evaluation, Seattle, Washington; Insitute for Health Metrics and Evaluation, Seattle, Washington; Washington University School of Medicine in St. Louis, St. Louis, MO

## Abstract

**Background:**

The COVID-19 pandemic highlighted the impact of viruses on lower respiratory tract diseases (LRTD); however, the global burden of viral LRTD is undefined. To bridge this gap, we estimated the global incidence of viral LRTD and hospitalizations in 2010–21.

**Figure 1.**
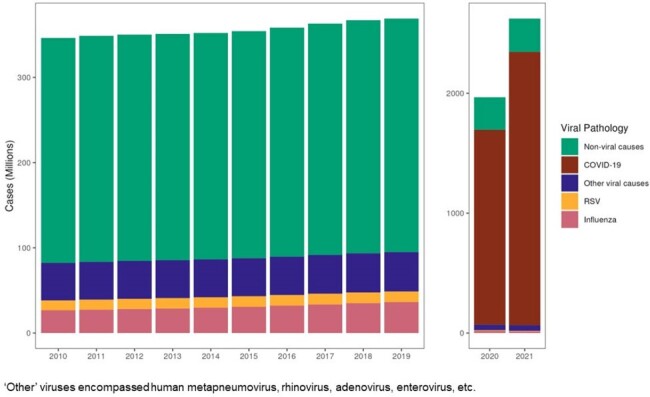
Global incident count of viral LRTD episodes by viral etiology (2010–2021). Non-viral causes are shown for comparison.

**Methods:**

We used the Global Burden of Diseases, Injuries, and Risk Factors 2021 (GBD) framework and a Bayesian meta-regression tool (DisMod-MR 2.1) to estimate the global incidence of viral LRTD. We defined viral LRTD as clinician-diagnosed pneumonia/bronchiolitis, capturing symptoms such as cough, difficulty breathing, fever, and chest discomfort. Viral LRTD were categorized by pathogen (SARS-Cov2; influenza; respiratory syncytial virus [RSV]; ‘other’). We estimated hospitalizations by applying a country-specific scalar, informed by inpatient admission data and a country-specific healthcare access and quality index from the GBD, on pathogen-specific estimates of LRTD for each country. In data sparse regions, estimates were generated by extrapolation.

**Figure 2.**
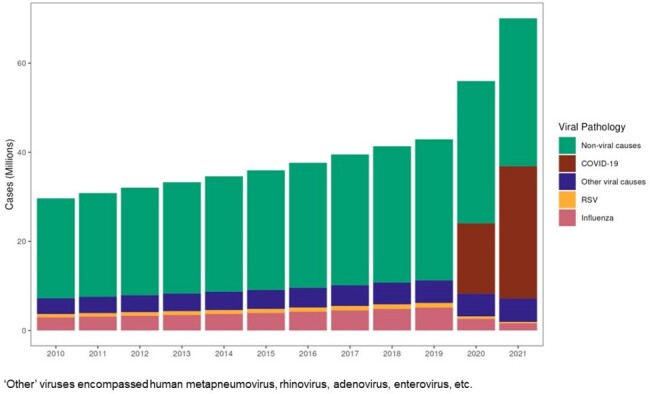
Global incident count of viral LRTD hospitalizations, by viral etiology (2010–2021). Non-viral causes are shown for comparison.

**Results:**

Average global incidence (per 100,000 person-years [PY]) of viral-associated LRTD episodes was 1,198 in 2010–19 and 25,716 in 2020–21. The number of viral LRTD cases was 880 million in 2010–19 and 4 billion in 2020–21. Average overall global incidence (per 100,000 PY) of viral LRTD hospitalizations was 170 (123 in 2010–19; 388 in 2020–21). The total number of hospitalizations was 90 million in 2010–19 and 61 million in 2020–21. Influenza and ‘other’ viruses accounted for most viral LRTD episodes (Figure 1) and hospitalizations in 2010–19 (Figure 2). Viral LRTD episode rates remained stable until 2019, while hospitalization rates increased. A decrease in influenza- and RSV-related episodes and hospitalizations was noted in 2020–21.

**Conclusion:**

Viral LRTD are common and incur substantial global healthcare burden. Year-on-year increases in severe cases requiring hospitalization, most recently compounded by the COVID-19 pandemic, are alarming. Given the previously underappreciated scale of the problem, increased surveillance and novel therapeutic options may help reduce the burden of viral LRTD.

**Disclosures:**

**Ekaterina Maslova, ScD**, AstraZeneca: Employee of AstraZeneca|AstraZeneca: Stocks/Bonds (Private Company) **Malin Fageras, PhD**, AstraZeneca: Full time employee|AstraZeneca: Stocks/Bonds (Private Company) **Lisa White, PhD**, AstraZeneca: Advisor/Consultant **Pratik Sinha, MBChB/PhD**, AstraZeneca: Advisor/Consultant|Prenosis Inc: Board Member

